# Enhanced Immunity and Infection Resistance in Mice Through Co-Expression of Porcine IL-3, IL-7, and IL-15 Fusion Molecules in *Yarrowia lipolytica*

**DOI:** 10.3390/biology14040366

**Published:** 2025-04-02

**Authors:** Junjie Peng, Linhan Zhang, Jiangling Li, Xuebin Lv, Rui Liu, Jianlin Chen, Gang Wang, Rong Gao

**Affiliations:** 1 College of Life Science, Sichuan University, Chengdu 610065, China; pengjunjie93@163.com (J.P.); linhanzhang@outlook.com (L.Z.); 2 National Engineering Research Center for Biomaterial, Sichuan University, Chengdu 610065, China; wgang@scu.edu.cn; 3 Sichuan Animal Science Academy, Chengdu 610066, China; lvxuebin@21cn.com (X.L.); liuruicau@126.com (R.L.); 4 School of Laboratory Medicine, Collaborative Innovation Center of Sichuan for Elderly Care and Health, Chengdu Medical College, Chengdu 610500, China; chenjianlin@cmc.edu.cn

**Keywords:** porcine IL-3, IL-7, IL-15, fusion expression, immunity, mice

## Abstract

China’s livestock industry grapples with challenges posed by infectious diseases and the misuse of antibiotics, resulting in a heightened risk of drug-resistant pathogens. This study explored the immunomodulatory effects of co-expressing porcine interleukin 3, 7, and 15 in *Yarrowia lipolytica*, denoted as Po1h-IL-3/7/15. A 42-day experiment involving mouse immunization and pathogen challenge was conducted, during which in vivo assessments of antibodies, immune-related cells, and gene expression were detected following oral administration of Po1h-IL-3/7/15. Immunological alterations in mice were analyzed using flow cytometry, qRT-PCR, ELISA, and HE staining. Notably, the serum IgG and intestinal sIgA levels in the Po1h-IL-3/7/15 group were substantially elevated compared to the control groups (*p* < 0.01), so were the contents of IL-7, IL-15, IFN-γ, IL-22, IL-23, and TNF-α. Furthermore, there was a marked increase in naïve T cells and central memory T cells, accompanied by a significant decrease in regulatory T cells in peripheral blood. Post-challenge with *Staphylococcus aureus* or *Salmonella typhimurium*, the expression levels of BD2, IL-1β, IL-8, Jak1, RegⅢ, S100A8, STAT1, and TNF-α genes in the intestines of the Po1h-IL-3/7/15 group were markedly higher than those in the control groups (*p* < 0.01). Following the challenges, the survival rate of the Po1h-IL-3/7/15 group was 100%, a significant increase compared to the 20% and 40% survival rates observed in the control groups (*p* < 0.05). These results confirm that IL-3/7/15 significantly boosts innate immunity, humoral and cell-mediated immune responses, and intestinal mucosal immunity in mice, enhancing resistance to bacterial infections and exhibiting potent protective effects.

## Simple Summary

Animal infectious diseases have caused significant disruptions to global livestock production. This study delved into the immunomodulatory impacts of co-expression of porcine interleukin 3, 7, and 15 in *Yarrowia lipolytica*, designated as Po1h-IL-3/7/15. The experimental regimen encompassing mouse immunization and pathogen challenge was executed, where in vivo evaluations of antibodies, immune-related cells, and gene expressions were undertaken after oral administration of Po1h-IL-3/7/15. Remarkably, the serum IgG and intestinal sIgA concentrations in the Po1h-IL-3/7/15 group rose considerably compared to the control groups, alongside heightened levels of IL-7, IL-15, IFN-γ, IL-22, IL-23, and TNF-α. Moreover, there was a notable surge in naïve T cells and central memory T cells, accompanied by a significant decline in regulatory T cells in peripheral blood. Following the challenge with *Staphylococcus aureus* or *Salmonella typhimurium*, the expression levels of innate immunity, Jak1 and STAT1 genes in the intestines of the Po1h-IL-3/7/15 group were substantially elevated compared to the control groups. Post-challenge, the survival rate in the Po1h-IL-3/7/15 group was 100%, marking a significant increase from the 20% and 40% survival rates observed in the control groups. These findings affirm that IL-3/7/15 potently enhances innate immunity, humoral and cell-mediated immune responses, and intestinal mucosal immunity in mice, ultimately bolstering resistance to bacterial infections and exhibiting robust protective efficacy.

## 1. Introduction

Animal infectious diseases have wrought havoc on global livestock. Furthermore, the imprudent use of antibiotics in animal farming has given rise to antibiotic-resistant pathogens, which have presented a formidable obstacle to contemporary human healthcare systems [1]. Consequently, establishing a modern framework that seamlessly integrates the prevention and treatment of animal infectious diseases, alongside the development of food or feed additives that bolster animal health and immunity, is paramount for ensuring the enduring prosperity of the global livestock industry.

Cytokines rank among the most potent immunomodulatory agents, playing pivotal roles in the differentiation, development, maturation, and activation of diverse immune cells [2]. These small, soluble proteins, secreted by immune cells and non-immune cells, serve as crucial intermediaries in intercellular communication [3]. Cytokines can function as vaccine adjuvants or anti-infective immune molecules, potentially stepping in for antibiotics to elevate the immune system’s capacity to fend off and combat infectious diseases [4]. Traditional veterinary vaccines frequently utilize oil-based adjuvants, which are hampered by side effects and localized adverse reactions. In stark contrast, cytokines exhibit high efficacy in modulating immune responses and demonstrate favorable safety profiles, rendering them appealing as prospective vaccine adjuvants. For pigs, IL-1β and IL-6 can stimulate lymphocyte proliferation, immunoglobulin production, hematopoiesis, and NK cell activity [5]. Studies by D.N. Reddy et al. revealed that pigs vaccinated with a *Streptococcus suis* vaccine augmented with recombinant bovine IL-1β exhibited milder clinical manifestations and zero fatalities upon infection compared to pigs administered the vaccine alone [6]. Likewise, Zhang Linghua et al. observed that the pairing of IL-6 gene and CpG sequences as adjuvants amplified the immune response of neonatal pigs to a pseudorabies attenuated vaccine, enhancing both humoral and cellular immunity [7]. Other cytokines, including IL-2, IL-4, IL-12, IL-18, IFN-γ, and GM-CSF, have also been explored as vaccine adjuvants or anti-infective immune molecules [5].

The synergistic activity of multiple cytokines represents a prevalent characteristic in biological processes, wherein their interactions render the regulation of nearly all physiological processes exceedingly intricate. For instance, IL-1, IL-2, and IL-6 collaborate in activating and functioning T cells; IL-3, IL-4, IL-5, and IL-6 are pivotal for B cell proliferation and differentiation, and IL-1 and IL-6 co-regulate acute-phase responses and superoxide dismutase expression [8]. Given the superior impact of multi-cytokine interactions compared to individual cytokines, ongoing research focuses on exploring the combination of various cytokines as immunopotentiators or anti-infective agents. For example, Jeffrey D. Ahlers et al. reported that combining GM-CSF, IL-12, and TNF-α with a peptide vaccine in mice shielded them against the HIV envelope protein gp160 and synergistically stimulated the production of cytotoxic T lymphocytes [9]. Tang Deyuan et al. constructed eukaryotic expression vectors for porcine IL-2 and IL-4, co-administering them with the ORF5 DNA vaccine for porcine reproductive and respiratory syndrome virus, thereby markedly enhancing antibody titers, neutralizing antibody levels, and specific T lymphocyte proliferation [10].

IL-3, produced by T cells, macrophages, stromal cells, NK cells, mast cells, and eosinophils, serves as a multi-lineage hematopoietic growth factor crucial for early hematopoiesis. In concert with erythropoietin, GM-CSF, and G-CSF, it prompts the production of erythroid and granulocyte–macrophage lineages, respectively [11]. Additionally, IL-3 synergizes with TNF-α to augment the proliferation of CD34^+^ progenitor cells and intensifies IL-2-induced B cell proliferation and differentiation. Early studies by Hawley R. J. et al. on the cloning and functional analysis of porcine IL-3 (pIL-3) revealed species specificity, significantly stimulating porcine bone marrow cell proliferation with minimal effects on primate cells [12]. Marion Andrew et al. further demonstrated that the pIL-3 gene or protein could amplify the protective efficacy of DNA vaccines against classical swine fever virus, identifying it as a potential novel adjuvant molecule [13].

IL-7, also known as pre-B cell growth factor or lymphopoietin 1, is indispensable for the survival, proliferation, and development of naïve and memory T cells, NK cells, and B cells. IL-7 signaling is crucial for coordinating thymocyte proliferation, differentiation, and T cell receptor α chain rearrangement. Studies on IL-7 and IL-7Rα gene knockout mice have emphasized its significance in maintaining the equilibrium of T and B cell development in vivo [14]. Satoshi Ueha et al. cloned the porcine IL-7 (pIL-7) cDNA from neonatal pig intestinal epithelial cells and examined its expression distribution across various tissues, finding expression in the intestine, thymus, kidney, and skin, but absent in the heart [15].

IL-15, produced by both non-immune (keratinocytes and skeletal muscle cells) and immune (monocytes and activated CD4^+^ T cells) cells, is induced in response to innate immune signals. The IL-15 receptor (IL-15R) comprises the IL-15Rα chain, IL-2Rβ chain, and γc, with IL-15 exhibiting a comparable capacity to IL-2 in stimulating the proliferation of T cells, NK cells, and innate lymphoid cells (ILCs) [16]. IL-15 plays a pivotal role in modulating the proliferation and activation of phytohemagglutinin-activated peripheral blood T cells, bolstering their ability to express IFN-γ and IL-4 and inducing cytotoxic T cell activity [17]. In activated B cells, IL-15 fosters proliferation and, in conjunction with CD40L, triggers IgG production. IL-3 and IL-7 function as colony-stimulating factors, inducing the generation of erythrocytes, granulocytes, and macrophages during hematopoiesis. IL-7 and IL-15 serve as lymphocyte growth factors, synergistically enhancing the proliferation of related cells and can further induce the differentiation of Th0 cells into Th1, Th2, and Th17 subtypes [18]. The co-expression of these three cytokines harnesses the benefits of multi-cytokine interactions, potentially yielding synergistic effects.

## 2. Materials and Methods

### 2.1. Construction of Self-Cloning Shuttle Vector pINA1297-IL-3/7/15

The genetic sequences of porcine interleukin 3 (IL-3, Sequence ID: NM_000588.4), 7 (IL-7, Sequence ID: NM_214135.2), and 15 (IL-15, Sequence ID: NM_214390.1) were acquired from GenBank. These sequences were integrated using two linker sequences, consisting of a flexible peptide GSG and a foot-and-mouth disease virus self-splicing 2A short peptide (P2A), to generate a fusion gene (IL-3_Linker_IL-7_Linker_IL-15). Additionally, a hexahistidine tag (6 × HisTag) was appended to the 3′ ends of each gene component to enable efficient purification. The pertinent sequence details for each constituent of the fusion gene are delineated in Table 1 below. Subsequently, employing gene synthesis services (Nanjing GenScript Biotechnology Co., Ltd., Nanjing, China), the fusion gene fragment was synthesized and integrated into the self-cloning shuttle vector pINA1297. The resultant recombinant shuttle vector was designated as pINA1297-IL-3/7/15.

### 2.2. Construction of Recombinant Yarrowia lipolytica Po1h-pINA1297-IL-3/7/15

Extract pINA1297-IL-3/7/15 recombinant plasmid according to the instructions of E.Z.N.A.^®^ Plasmid DNA Mini Kit I (Omega Bio-Tek, Norcross, GA, USA). pINA1297-IL-3/7/15 was linearized using Not I (Thermo, Waltham, MA, USA), and then, *Yarrowia lipolytica* Po1h competent cell was prepared according to the instructions of the Frozen-EZ Yeast Transformation II Kit (Zymo Research, Freiburg im Breisgau, Germany), a chemical transformation method. Competent cells were transformed with pINA1297-IL-3/7/15 linearized fragment. Recombinants (Po1h-pINA1297-IL-3/7/15 designated as Po1h-IL-3/7/15) were screened through uracil-deficient SD plates (Shanghai Biotech TakaRa, Shanghai, China). According to the above method, the empty vector pINA1297 was transformed into *Yarrowia lipolytica* Polh (designated as Polh-pINA1297), which was used as a control.

### 2.3. Expression and In Vitro Activity Analysis of Fusion IL-3/7/15 in Yarrowia lipolytica

The strains Po1h- IL-3/7/15 and Po1h-pINA1297 were individually cultured in 100 mL YPD liquid medium (consisting of 10 g/L yeast extract, 20 g/L tryptone, and 20 g/L glucose) within 250 mL shake flasks. Cultivation was performed with continuous shaking in a temperature-controlled air bath at 28 °C and 200 rpm for a duration of 48 h. Subsequently, the fermentation broth underwent ultrasonic lysis (using a power of 200 W, with ultrasonic bursts lasting 3 s and intervals of 10 s, repeated 30 times). The supernatant was collected following centrifugation at 10,000 rpm at 4 °C, and the expression levels of the recombinant proteins were determined, according to the instructions provided with the Porcine Interleukin 3 (IL-3) ELISA Kit, Porcine Interleukin 7 (IL-7) ELISA Kit, and Porcine Interleukin 15 (IL-15) ELISA Kit, all sourced from CUSABIO, Wuhan, China.

An amount of 5 mL of peripheral blood was drawn from the anterior vena cava of pigs and anticoagulated with EDTA-2K. Pig lymphocytes were subsequently isolated following the protocol provided by the pig peripheral blood lymphocyte isolation solution KIT (Tianjin Haoyang Huake Biotechnology Co., Ltd., Tianjin, China). The isolated cells were diluted with a complete 1640 culture medium (comprising 10% calf serum, 100 µg/mL ampicillin, and 100 µg/mL streptomycin) to adjust a final cell concentration of 2 × 10^6^ cells/mL. These cells were then seeded into 10 cm cell culture dishes at a volume of 10 mL per dish. Sigma-Aldrich^®^ Con A (L7647, Merck KGaA, Darmstadt, Germany) was added to a final concentration of 10 µg/mL for cellular stimulation, followed by incubation at 37 °C in a 5% CO_2_ culture incubator for 24 h. After 24 h of incubation, porcine lymphoblasts in the culture dishes were harvested into clean centrifuge tubes, centrifuged at 1500 rpm for 15 min to pellet the cells, washed twice with a complete 1640 culture medium, and centrifuged again at 1500 rpm for 15 min. The cells were then resuspended in a 1640 culture medium to achieve a cell concentration of approximately 6 × 10^6^ cells/mL. Subsequently, 100 µL of the target cells (porcine lymphoblasts) were mixed with an equal volume of Po1h-pINA-IL-3/7/15 or Po1h-pINA1297, respectively, in the experimental wells of a 96-well plate. The plate was then cultured in a 37 °C, 5% CO_2_ cell culture incubator for 48 h. Following the incubation period, 10 µL of CCK8 (BMU106-CN, Abbknie, Wuhan, China) was added to each well, gently mixed, and further incubated for 2 h. Subsequently, the plate was removed from the incubator, and the optical density at 450 nm (OD_450_) of each well was measured using a Bio-Reader680 (Bio-Rad Laboratories, Inc., Hercules, CA, USA).

### 2.4. In Vivo Activity in Mice

#### 2.4.1. Animal Treatment

Thirty 4–5-week-old female Kunming mice were randomly divided into three groups: PBS control, Po1h-pINA1297, and Po1h-IL-3/7/15. Each group received oral administration every three days for a total of 10 doses. The experimental groups received 4 × 10^8^ CFU/100 µL of respective fermentation broths, while the control group received 100 µL of Phosphate buffered saline (PBS) solution. Mice in all groups were weighed weekly to monitor any changes in body weight over the four-week treatment period. After 28 days, the mice (5 mice per group) were challenged with either 1.0 × 10^9^ CFU *Salmonella typhimurium* (ATCC 14028) or 1.0 × 10^9^ CFU *Staphylococcus aureus* (ATCC 25923) through oral administration once every other day for a total of three times. Survival rates and health status were monitored daily. Peripheral blood samples were collected from the tail vein of mice on days 7, 14, 21, and 28 post-administration and three days post-pathogen challenge. The animal experiments in this study were approved by the Animal Ethics Committee (ACE) of the Animal Experiment Center of Sichuan University (license: SYXK-Chuan-2019-172). All experimental procedures and animal welfare standards strictly followed the animal management guidelines of Sichuan University.

#### 2.4.2. Changes in Immune-Related Genes in Mouse Peripheral Blood

Total RNA was extracted from peripheral anticoagulant blood of mice on days 14 and 28 post-administration and on the third day post-challenge, and cDNA was obtained by reverse transcription. Fluorescence quantitative PCR was used to detect immune-related genes (IL-7, IL-15, IL-22, IL-23, IFN-γ and TNF-α). Primers used to detect target genes in mice are shown in Table 2.

#### 2.4.3. Changes in Total IgG in Mouse Plasma

An amount of 200 μL of EDTA anticoagulated peripheral blood from the tail vein of mice was obtained on the 7th, 14th, and 28th day post-administration and the 3rd day post-challenge. Total IgG levels were measured using an ELISA kit according to the manufacturer’s instructions (RX202736M, Ruixin Biotechnology, Quanzhou, China).

#### 2.4.4. Changes in Immune-Related Genes in Mouse Small Intestine Tissue

An amount of 25 mg of mouse small intestinal tissue was collected post-challenge, ground with liquid nitrogen, and total RNA was extracted and reverse transcribed into cDNA following TransScript One-step gDNA Removal and cDNA Synthesis SuperMix protocol, and fluorescence quantitative PCR was used to detect Jak-1, STAT1, IL-1β, IL-8, BD2, S100A8, RegIII, and TNF-α genes. The primers used are shown in Table 3.

#### 2.4.5. Secretory Immunoglobulin A (sIgA) Levels in Mouse Feces 

Fresh feces samples (0.5 g) were collected on day 28 post-administration and the 3rd day post-challenge. A total of 0.01 M PBS containing 0.05 M EDTA buffer at 4 mL/g was added to suspend the samples; then, they were shaken on ice for 15 min and centrifuged at 10,000× *g* for 5 min at 4 °C. The supernatant was frozen at −80 °C until detection. The fecal sIgA content was detected according to the mouse secretory immunoglobulin A (sIgA) quantitative detection kit (ELISA) instruction manual (RX-G202950M, Ruixin Biotechnology, Quanzhou, China).

#### 2.4.6. Histological Changes in Mouse Intestinal Tissue Post-Challenge

Post-challenge, small intestinal tissues were obtained from mice in each group and stained with hematoxylin and eosin (H & E) to observe and measure villus height, crypt depth, and intestinal wall thickness to evaluate the structure and function of the small intestine.

#### 2.4.7. Flow Cytometry Analysis of Immune Cell Changes in Peripheral Blood of Mice

Peripheral blood mononuclear cells (PBMCs) on days 7, 14, and 28 post-administration were isolated from whole blood samples using Ficoll–Paque density gradient centrifugation (Cytiva, Marlborough, MA, USA). Isolated PBMCs were resuspended in staining buffer (PBS containing 2% fetal bovine serum and 0.1% sodium azide) at a concentration of 1 × 10^6^ cells/mL. Cells were then incubated with fluorochrome-conjugated monoclonal antibodies specific for markers (Tube 1 for B cells: CD19-BV510, CD27-PE, CD38-BV421, IgD-APC and Fixable Viability Stain 700, Tube 2 for T cells: CD3-APC-Cy7, CD4-FITC, CD8a-BV510, CD44-PE-Cy7, CD62L-BUV395, CD25-BV421, FOXP3-PE, Fixable Viability Stain 700) for 30 min at 4 °C in the dark. After surface maker staining, T cells were further fixed and permeabilized by BD PharmingenTM Transcription-Factor Buffer Set for intracellular staining of FOXP3. Following incubation, cells were washed twice with a staining buffer to remove unbound antibodies. Stained cells were resuspended in 500 µL of staining buffer and analyzed using a BD FACSymphonyTM A5 Cell Analyzer (BD Biosciences, San Jose, CA, USA). Compensation was performed using BD CompBeads (BD Biosciences) stained individually with each fluorochrome. Data were acquired using FACSDiva software Version 6 (BD Biosciences) and analyzed with FlowJo v10.8.1 software (Tree Star Inc., Ashland, OR, USA). Lymphocyte populations were gated based on forward and side scatter properties. Further gating was performed to identify specific cell subsets: CD4^+^/CD62L^+^/CD44^+^ central memory T cells (TCM), CD4^+^/CD62L^−^/CD44^+^ effector memory T cells (TEM), CD4^+^/FoxP3^+^/CD25^+^ regulatory T cells (Treg), CD4^+^/FoxP3^−^/CD25^+^ effector T cells (Teff), CD19^+^/CD27^+^/CD38^+^ plasma cells, CD19^+^/CD27^+^/IgD^+^ non-switched memory B cells, and CD19^+^/CD27^+^/IgD^−^ class-switched memory B cells. The percentage of each cell subset was calculated relative to the total lymphocyte population.

### 2.5. Statistical Analysis

Data were analyzed using GraphPad Prism software (version 9.5.1). Comparisons among groups were made using two-way ANOVA followed by Tukey’s multiple comparison test. Differences were considered significant at *p* < 0.05.

## 3. Results

### 3.1. Screening, Verification, and Functional Expression Analysis of Recombinant Yarrowia lipolytica Po1h-IL-3/7/15

**Figure 1:**
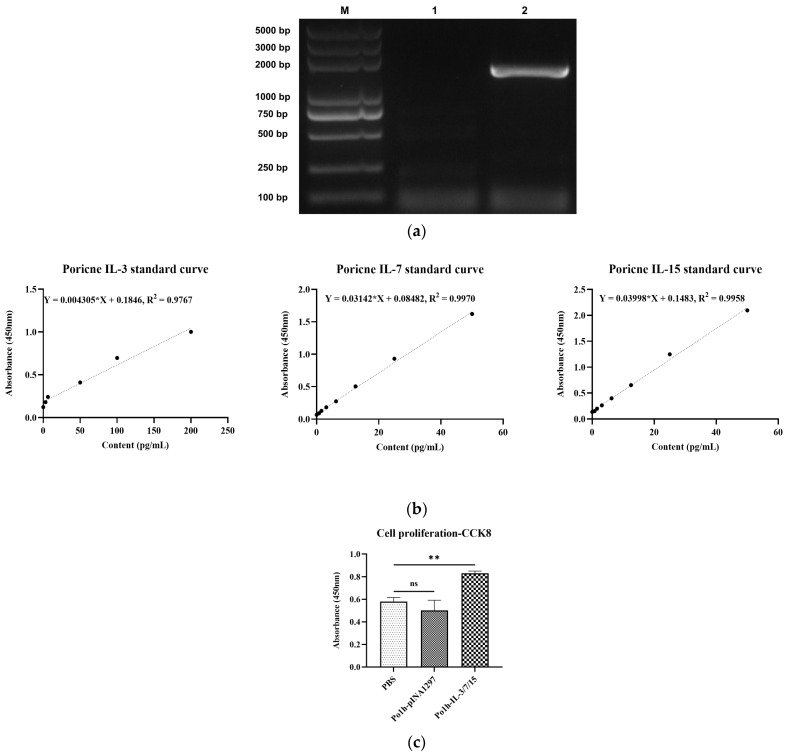
Agarose gel electrophoresis for colony PCR verification of recombinant *Yarrowia lipolytica* Po1h-IL-3/7/15 (**a**). Gel electrophoresis image showing the PCR product of approximately 1800 bp in lane 2, confirming the integration of the fusion gene fragment into the genome. ELISA analysis of recombinant protein expression in Po1h-IL-3/7/15 supernatant (**b**). Bar graph depicting the concentrations of standard porcine IL-3, IL-7, and IL-15. CCK-8 Assay results for cell proliferation stimulated by Po1h-IL-3/7/15 supernatant (**c**). Graph shows enhanced cell proliferation activity in the Po1h-IL-3/7/15 group compared to the PBS and Po1h-pINA1297 groups. ns: not statistically significant, **: *p* < 0.01.

Transformants were screened using a uracil-deficient SD medium, and single colonies were selected to identify positive bacterial transformants via colony PCR. Analysis using 1.5% agarose gel electrophoresis (Figure 1a) revealed that the colony PCR product in lane 2 contained a gene fragment of approximately 1800 bp. Subsequent sequencing of the band confirmed that the sequence was 100% identical to the designed sequence, indicating the successful integration of the fusion gene fragment into the genome. Thus, the recombinant Po1h-IL-3/7/15 strain was successfully constructed. To assess the expression of recombinant proteins, ELISA kits, specific for porcine Interleukin 3 (IL-3), porcine Interleukin 7 (IL-7), and porcine Interleukin 15 (IL-15) (CUSABIO, China) were used to analyze the supernatant of the recombinant yeast. As shown in Figure 1b and Table 4, the target proteins were detected in the supernatant of the recombinant yeast Po1h-IL-3/7/15, with concentrations of porcine IL-3, IL-7, and IL-15 measured at 7.844 ng/mL, 11.300 ng/mL, and 0.440 ng/mL, respectively. No target protein expression was observed in the supernatant of the recombinant yeast Po1h-pINA1297. Following stimulation and culture of porcine cells with PBS, Po1h-pINA1297 fermentation supernatant, and Po1h-IL-3/7/15 fermentation supernatant, cell proliferation was assessed using the CCK-8 assay. The results, shown in Figure 1c, indicate that the Po1h-IL-3/7/15 group exhibited significantly higher proliferation activity compared to the PBS group and Po1h-pINA1297 group (*p* < 0.01).

### 3.2. Immunoglobulin Response in Mice Immunized with Recombinant Po1h-IL-3/7/15

**Figure 2:**
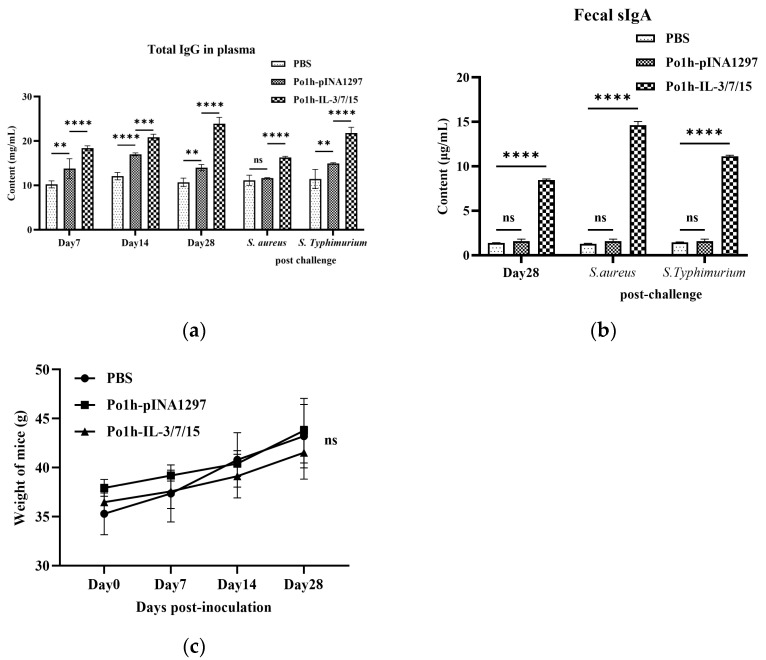
Plasma IgG levels in mice immunized with Po1h-IL-3/7/15 (**a**). Graph illustrating total plasma IgG levels in the Po1h-IL-3/7/15 group compared to the PBS and Po1h-pINA1297 groups. Fecal sIgA Levels in mice immunized with Po1h-IL-3/7/15 (**b**). Graph showing fecal sIgA levels in the Po1h-IL-3/7/15 group compared to the PBS and Po1h-pINA1297 groups. Body weight changes in mice immunized with Po1h-IL-3/7/15 (**c**). Graph depicting the body weight changes among different groups, indicating no significant differences (ns: not statistically significant, **: *p* < 0.01, ***: *p* < 0.001, ****: *p* < 0.0001).

Peripheral blood plasma and fecal samples were collected from each group of mice at different time points to measure IgG levels in plasma and sIgA levels in feces using ELISA. As shown in Figure 2a,b, the total plasma IgG and fecal sIgA levels in the Po1h-IL-3/7/15 group were significantly higher than those in the PBS and Po1h-pINA1297 groups both before and after challenge (*p* < 0.01). The differences in body weight among the groups at four time points were not statistically significant (*p* > 0.05), indicating that the recombinant yeast possesses reliable biosafety (Figure 2c). This suggests that the recombinant yeast effectively enhances both systemic and mucosal immune responses. The elevated IgG levels indicate improved humoral immunity, while increased sIgA reflects heightened intestinal mucosal immunity.

### 3.3. Flow Cytometric Analysis of Lymphocyte Subtypes After Immunization with Po1h-IL-3/7/15 in Mice

**Figure 3:**
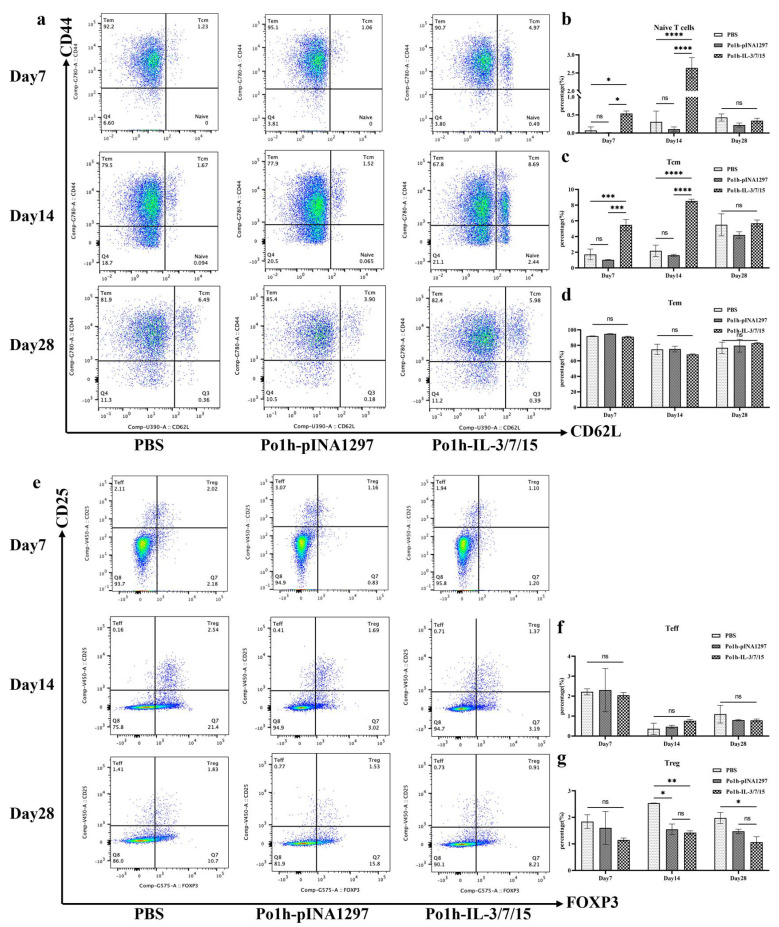
Flow cytometry scatter plots of T lymphocyte subtypes in mice post-immunization (**a**,**e**). Scatter plots showing Naïve T cells, Tcm, Tem, Teff, and Treg subtypes in the peripheral blood of mice. Quantitative analysis of T lymphocyte subtypes in mice immunized with Po1h-IL-3/7/15 (**b**–**d**,**f**,**g**). Graphs illustrating the levels of Naïve T cells, Tcm, Tem, Teff, and Treg cells in the Po1h-IL-3/7/15 group compared to control groups. ns: not statistically significant, *: *p* < 0.05, **: *p* < 0.01, ***: *p* < 0.001, ****: *p* < 0.0001.

At various time points, peripheral blood from the mice was labeled with fluorescent antibodies and analyzed by flow cytometry to assess the changes in T and B lymphocyte subtypes across the PBS, Po1h-pINA1297, and Po1h-IL-3/7/15 groups. Figure 3a presents flow cytometry scatter plots showing the immunophenotyping of Naïve T cells, central memory T cells (Tcm), and effector memory T cells (Tem) in the peripheral blood of the mice, while Figure 3e displays scatter plots for the immunophenotyping of effector T cells (Teff) and regulatory T cells (Treg). As shown in Figure 3b,c, the levels of naïve T cells and Tcm in the Po1h-IL-3/7/15 group were significantly or highly significantly elevated compared to the PBS and Po1h-pINA1297 groups on days 7 and 14 (*p* < 0.01), but no significant differences were observed between the experimental and control groups by day 28 (*p* > 0.05). In Figure 3d,f, there were no significant differences in Tem and Teff counts between the experimental and control groups at any time point (*p* > 0.05). As shown in Figure 3g, on days 14 and 28, Treg cell counts in the Po1h-pINA1297 and Po1h-IL-3/7/15 groups were significantly lower than those in the PBS group (*p* < 0.05). This indicates that the recombinant yeast promotes the activation and expansion of T cells, which are crucial for initiating adaptive immunity. The early decrease in regulatory T cells might temporarily reduce immune suppression, enhancing responsiveness during immunization.

### 3.4. Flow Cytometric Assessment of B Lymphocyte Subpopulations in Peripheral Blood Post-Immunization

**Figure 4:**
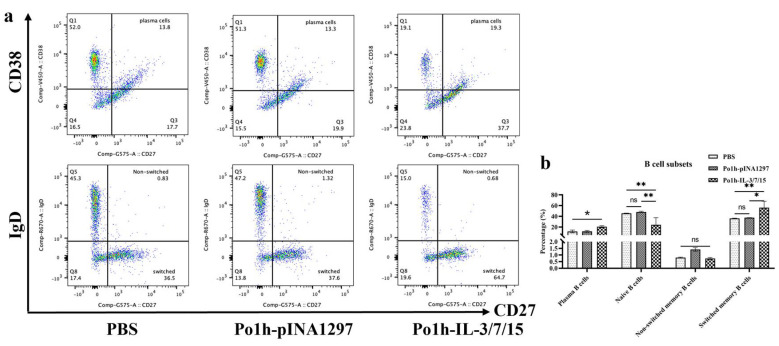
Flow cytometry scatter plots of B lymphocyte subpopulations in mice post-immunization (**a**). Scatter plots showing B lymphocyte subpopulations in peripheral blood on day 28. Quantitative analysis of B lymphocyte subpopulations in mice immunized with Po1h-IL-3/7/15 (**b**). Graphs showing the levels of plasma cells, switched memory B cells, non-switched memory B cells, and naïve B cells in the Po1h-IL-3/7/15 group. ns: not statistically significant, *: *p* < 0.05, **: *p* < 0.01.

Figure 4a shows flow cytometry scatter plots of B lymphocyte subpopulations in peripheral blood on day 28. In the Po1h-IL-3/7/15 group, the levels of plasma cells and switched memory B cells were significantly higher than those in the PBS and Po1h-pINA1297 groups (*p* < 0.05). However, there were no significant differences in the levels of non-switched memory B cells between the experimental and control groups (*p* > 0.05). The levels of naïve B cells in the Po1h-IL-3/7/15 group were significantly lower than those in the Po1h-pINA1297 and PBS groups (*p* < 0.01). This result suggests that the recombinant yeast promotes B cell maturation and differentiation into antibody-producing cells, aligning with the observed increase in IgG and sIgA levels.

### 3.5. Quantitative PCR Analysis of Immune-Related Gene Expression in PBMC and Small Intestinal Tissues

**Figure 5:**
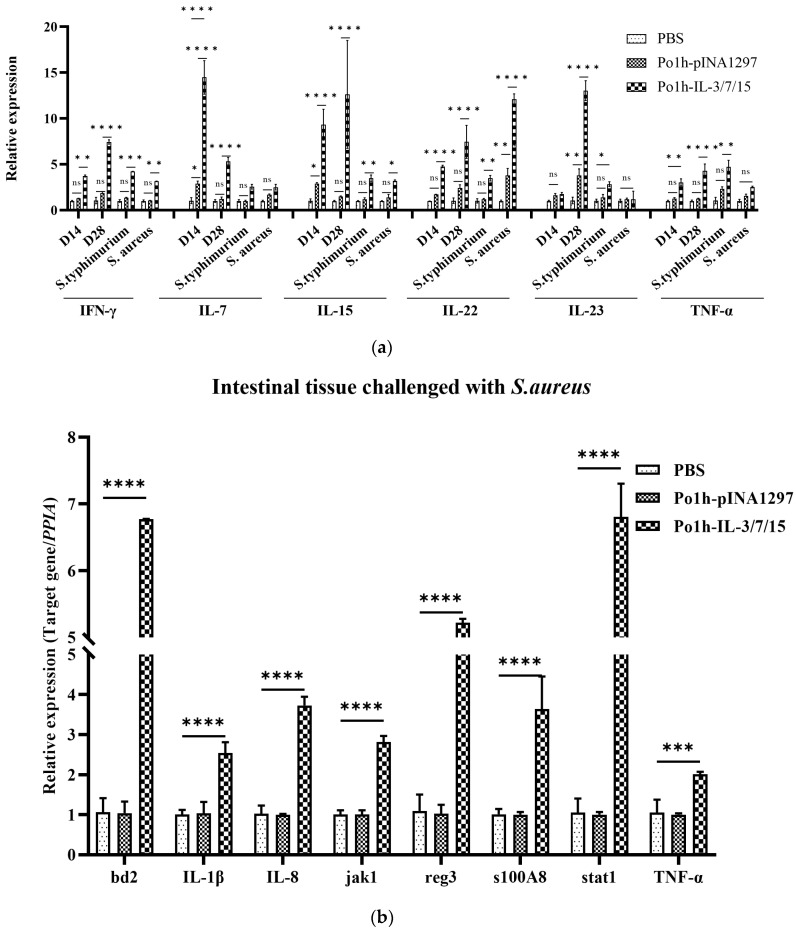
Quantitative PCR analysis of immune-related gene expression in PBMCs post-immunization and challenge (**a**). Expression levels of IL-7, IL-15, IFN-γ, IL-22, IL-23, and TNF-α in the Po1h-IL-3/7/15 group compared to the PBS group at different time points and after *S. Typhimurium* or *S. aureus* challenge. Quantitative PCR analysis of immune-related gene expression in small intestinal tissues post-*S. aureus* challenge (**b**). Bar graph showing significantly higher expression levels of BD2, IL-1β, IL-8, Jak1, RegIII, s100A8, STAT1, and TNF-α in the Po1h-IL-3/7/15 group compared to PBS and Po1h-pINA1297 groups (*p* < 0.01). ns: not statistically significant, *: *p* < 0.05, **: *p* < 0.01, ***: *p* < 0.001, ****: *p* < 0.0001.

The Po1h- IL-3/7/15 group showed significantly higher expression of IL-7 on days 14 and 28, IL-15, IFN-γ, and IL-22 at all time points (*p* < 0.01) compared to the control groups. IL-23 expression was significantly elevated on day 28 and after the *S. Typhimurium* challenge (*p* < 0.01). TNF-α expression peaked under *S. Typhimurium* challenge and was higher on days 14 and 28 (*p* < 0.01) compared to the control groups (Figure 5a).

After the challenge with *Staphylococcus aureus*, small intestinal tissues from each group of mice were collected for quantitative PCR analysis to assess changes in the expression of immune-related genes. As shown in Figure 5b, the expression levels of BD2, IL-1β, IL-8, Jak1, RegIII, s100A8, STAT1, and TNF-α in the Po1h-IL-3/7/15 group were significantly higher than those in the PBS and Po1h-pINA1297 groups (*p* < 0.01). No significant differences in the expression of these genes were observed between the PBS and Po1h-pINA1297 groups (*p* > 0.05), indicating that the empty vector-carrying *Yarrowia lipolytica* Po1h strain does not affect the expression levels of immune-related genes in the small intestinal tissues of mice following *S. aureus* challenge. Upregulation of these genes highlights the activation of innate and adaptive immune pathways. Enhanced intestinal gene expression suggests robust mucosal immunity, providing protection against bacterial pathogens.

### 3.6. Histological Analysis of Small Intestinal Structure Following Bacterial Challenge

**Figure 6:**
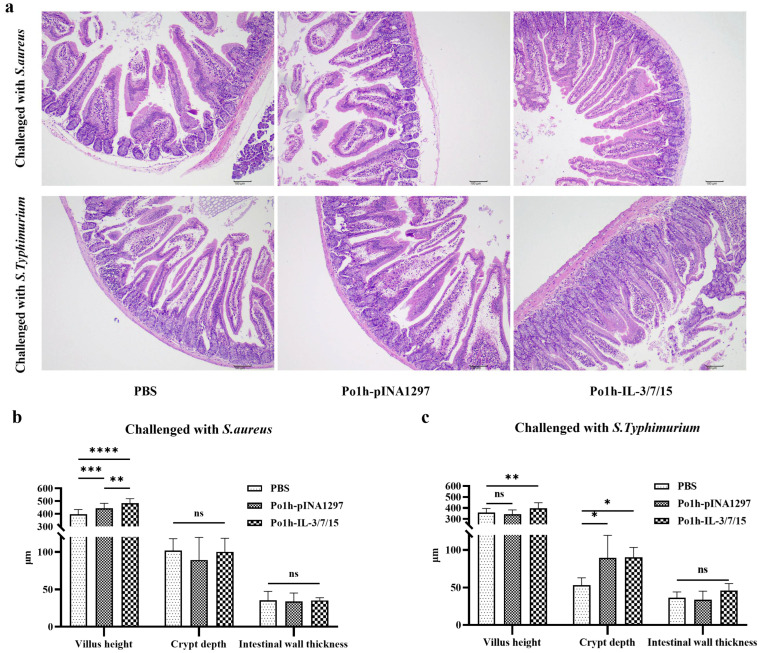
Histological analysis of small intestinal structure post-bacterial challenge (**a**). Images of H&E-stained small intestinal tissues showing the structure of villi, crypts, and intestinal wall thickness. Quantitative measurement of villus height, crypt depth, and intestinal wall thickness post-bacterial challenge (**b**,**c**). Bar graphs illustrate the significantly greater villus height and crypt depth in the Po1h-IL-3/7/15 group compared to PBS and Po1h-pINA1297 groups, with no significant differences in intestinal wall thickness. ns: not statistically significant, *: *p* < 0.05, **: *p* < 0.01, ***: *p* < 0.001, ****: *p* < 0.0001.

After the challenge, small intestinal tissues from each group of mice were collected and subjected to hematoxylin and eosin (H&E) staining to observe and measure villus height, crypt depth, and intestinal wall thickness, to assess the structure and function of the small intestine. The stained tissue sections are shown in Figure 6a, and three key metrics were quantified microscopically. As illustrated in Figure 6b,c, following the challenge with *S. aureus* and *S. Typhimurium*, the villus height in the small intestine of the Po1h-IL-3/7/15 group was significantly greater than that in the PBS and Po1h-pINA1297 groups (*p* < 0.01). No significant differences in intestinal wall thickness were observed between the experimental and control groups (*p* > 0.05). After the *S. Typhimurium* challenge, crypt depth in the Po1h-IL-3/7/15 and Po1h-pINA1297 groups was significantly greater than that in the PBS group (*p* < 0.05).

### 3.7. Survival Analysis Post-Bacterial Challenge

**Figure 7:**
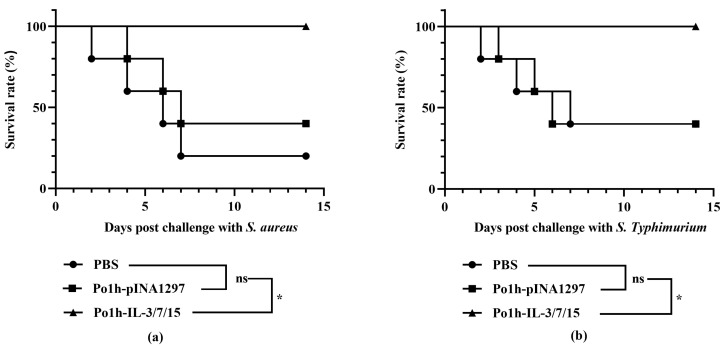
Survival analysis of mice post-*S. aureus* challenge (**a**). Kaplan–Meier survival curve showing a 100% survival rate in the Po1h-IL-3/7/15 group compared to a 20% survival rate in the PBS group and 40% in Polh1297 group. Survival analysis of mice post-*S. Typhimurium* challenge (**b**). Kaplan–Meier survival curve showing a 100% survival rate in the Po1h-IL-3/7/15 group compared to a 40% survival rate in the PBS group and Polh1297 group. ns: not statistically significant, *: *p* < 0.05.

Under both challenge conditions, the survival rate of mice in the Po1h-IL-3/7/15 group was significantly higher than that in the control groups (*p* < 0.05) (Figure 7). As shown in Figure 7a, two weeks after the *S. aureus* challenge, only 20% of the mice in the PBS group survived, whereas the Po1h-IL-3/7/15 group had a 100% survival rate. Similarly, as depicted in Figure 7b, two weeks after the *S.Typhimurium* challenge, the survival rate of mice in the PBS group was 40%, while the Po1h-IL-3/7/15 group maintained a 100% survival rate. This highlights the protective efficacy of the recombinant yeast in conferring resistance to bacterial infections.

## 4. Discussion

Following the successful construction of recombinant Po1h-IL-3/7/15, it is essential to evaluate the biological effects of the fusion molecule through immunological assays. It assesses the cellular and humoral immune responses in mice following oral administration, focusing on lymphocyte dynamics, antibody levels, and immune-related gene expression, providing evidence for the potential of the IL-3/7/15 fusion molecule as an immunostimulant.

Cytokines are known for their complex and multifaceted roles in regulating the immune system, often used to modulate various aspects of immune responses. IL-3 supports hematopoiesis and enhances the proliferation of progenitor cells, which can complement the lymphocyte growth-promoting effects of IL-7 and IL-15. IL-7 is critical for the survival and development of naïve and memory T cells, while IL-15 plays a pivotal role in activating and maintaining NK cells and memory T cells. Together, IL-7 and IL-15 have been shown to synergistically promote the proliferation and differentiation of T cells, including cytotoxic T lymphocytes, which are crucial for adaptive immunity. The co-expression of these cytokines could harness their combined effects, potentially leading to enhanced immune cell activation, proliferation, and differentiation. For instance, IL-7 and IL-15 might work together to sustain long-term memory T cells, while IL-3 could provide additional support by boosting the production of precursors of immune cells. Fluorescent quantitative PCR analysis of mouse peripheral blood mononuclear cells (PBMCs) revealed that IL-7 and IL-15 expression levels were upregulated in the Po1h-IL-3/7/15 group prior to challenge (Figure 5a). This upregulation, reflecting the biological effects of IL-7 and IL-15, correlated with an increase in T lymphocyte numbers. Additionally, IL-22 showed a marked increase in expression both before and after the challenge (Figure 5a). As a tissue-signaling cytokine, IL-22 plays a crucial role in protecting and regenerating barrier organs such as the skin, lungs, and gastrointestinal tract [19]. It also promotes the release of BD2, BD3, and S100 family peptides (including S100A7, S100A8, and S100A9) from keratinocytes, which are responsible for the release of Reg3β and Reg3γ in the gastrointestinal tract of mice [20,21]. This explains the upregulated expression of BD2, S100A8, and Reg3 protein genes observed in the fluorescent quantitative PCR analysis of mouse small intestine tissue (Figure 5b).

In T-cell-mediated adaptive immune responses, the development, differentiation, and proliferation of T lymphocytes are key indicators of cellular immunity. Flow cytometry analysis of mouse peripheral blood T cells revealed that the proportions of naïve T cells and central memory T cells (TCM) peaked on day 14 of immunization, with a significant increase in the Po1h -IL-3/7/15 group compared to the PBS group. Jens Geginat demonstrated that IL-7 alone has a limited ability to induce TCM or TEM proliferation and activation, but when used in combination with IL-15, it effectively promotes TEM proliferation and has a moderate effect on TCM [22]. These findings, combined with the flow cytometry results for TCM (Figure 3c), further indicate the favorable impact of Po1h-IL-3/7/15 on T-cell proliferation and activation. Throughout the 28-day immunization period, the proportion of regulatory T cells (Tregs) in the Po1h-IL-3/7/15 group remained consistently lower than in the PBS group (Figure 3g). Treg cells are known to downregulate IL-22 expression at the transcriptional level [23], with IL-22 being a direct target gene of the Foxp3 transcription factor, a key marker of Tregs [24]. This suggests that the persistently low proportion of Tregs during the immunization process may contribute to the stable high expression of IL-22. Since Tregs primarily function to negatively regulate immune responses to prevent excessive immune reactions, their sustained low proportion also implies an enhanced immune response in the Po1h -IL-3/7/15 group.

In B cell-mediated specific immune responses, the development, differentiation, and proliferation of B cells, along with the secretion of specific antibodies, are critical indicators of humoral immunity. IgG, a highly affine antibody, is primarily produced during secondary immune responses and is present at high concentrations throughout the body, serving as one of the major effector molecules in systemic humoral immune responses. The plasma IgG antibody levels in the Po1h-IL-3/7/15 group remained consistently high, around 15 mg/mL, both before and after the challenge (Figure 2a), indicating a significant enhancement of the humoral immune response. IgA, which accounts for only 10–15% of total serum immunoglobulins, predominantly exists in secretory form (sIgA) in mucosal areas such as the respiratory, gastrointestinal, and urogenital tracts, preventing pathogen colonization [25]. High-affinity IgA antibodies, which protect the intestinal mucosal surface from pathogenic microorganisms, are produced via a T-cell-dependent pathway. In contrast, low-affinity IgA antibodies are produced via a T-cell-independent pathway and limit commensal bacteria to the intestinal lumen through a process known as “immune exclusion” [26,27]. In mice inoculated with the fusion molecule, fecal sIgA levels reached 10 mg/mL, 4–5 times higher than those of the control groups (Figure 2b), clearly demonstrating enhanced immune function in the intestinal mucosal tissues. These results are closely in accordance with the better survival rate of mice inoculated with Po1h-IL-3/7/15 after challenge with *S. aureus* and *S. typhimurium* compared to the control groups.

## 5. Conclusions

These promising results proved that the recombinant *Yarrowia lipolytica* co-expressing pig IL-3/7/15 was successfully constructed and would inspire the Po1hpINA1297-IL- IL-3/7/15 to be developed into safe, effective, and economical immune molecules to facilitate the control of animal bacterial infections.

## Figures and Tables

**Table 1:** Fusion gene sequences.

Name	Sequences
GSG	GGA AGC GGA
P2A	GGA AGC GGA GCT ACT AAC TTC AGC CTG CTG AAG CAG GCT GGA GAC GTG GAG GAG AAC CCT GGA CCT
6 × HisTag	CAT CAT CAT CAT CAT CAT

**Table 2:** QPCR primers.

Gene	Sequence	Tm (°C)
PPIA-F	CATACAGGTCCTGGCATCTTGTC	59
PPIA-R	AGACCACATGCTTGCCATCCAG	
IL-7-F	CAGGAACTGATAGTAATTGCCCG	61.5
IL-7-R	CTTCAACTTGCGAGCAGCACGA	
IL-15-F	GTAGGTCTCCCTAAAACAGAGGC	58
IL-15-R	TCCAGGAGAAAGCAGTTCATTGC	
IL-22-F	GCTTGAGGTGTCCAACTTCCAG	58.5
IL-22-R	ACTCCTCGGAACAGTTTCTCCC	
IL-23-F	CATGCTAGCCTGGAACGCACAT	61.5
IL-23-R	ACTGGCTGTTGTCCTTGAGTCC	
IFN-γ-F	CAGCAACAGCAAGGCGAAAAAGG	52
IFN-γ-R	TTTCCGCTTCCTGAGGCTGGAT	
TNF-α-F	GGTGCCTATGTCTCAGCCTCTT	56
TNF-α-R	GCCATAGAACTGATGAGAGGGAG	

**Table 3:** QPCR primers.

Gene	Sequence	Tm (°C)
BD2-F	AGGAGCAGACCCAAGTCCTAT	59
BD2-R	GGAAAGTCCCAGTCCCTGTT	
IL-1β-F	TGGACCTTCCAGGATGAGGACA	59
IL-1β-R	GTTCATCTCGGAGCCTGTAGTG	
Jak1-F	CTGTCTACTCCATGAGCCAGCT	60
Jak1-R	CCTCATCCTTGTAGTCCAGCAG	
S100A8-F	CAAGGAAATCACCATGCCCTCTA	59.5
S100A8-R	ACCATCGCAAGGAACTCCTCGA	
RegIII-F	GGGTTACAAGGCTTATCGCTCC	59
RegIII-R	GGACACAAAGGAAGCCTCACCT	
IL-8-F	GGTGATATTCGAGACCATTTACTG	57
IL-8-R	GCCAACAGTAGCCTTCACCCAT	
STAT1-F	GCCTCTCATTGTCACCGAAGAAC	60
STAT1-R	TGGCTGACGTTGGAGATCACCA	
TNF-α-F	GGTGCCTATGTCTCAGCCTCTT	60
TNF-α-R	GCCATAGAACTGATGAGAGGGAG	

**Table 4:** The analysis of fusion interleukins in Po1h-IL-3/7/15 Supernatant.

Samples	OD_450_ (Dilution Fold)		
	Porcine IL-3	Porcine IL-7	Porcine IL-15
Po1h-IL-3/7/15	0.86 (50 × dilution)	1.86 (200 × dilution)	0.5 (50 × dilution)
	7.844 ng/mL	11.300 ng/mL	0.440 ng/mL

## Data Availability

Data are contained within the article.

## Abbreviations

The following abbreviations are used in this manuscript:

IL	Interleukin
*S. typhimurium*	Salmonella typhimurium
NK	Natural killer cell
PBMC	Peripheral blood mononuclear cell
*S. aureus*	Staphylococcus aureus
Teff	Effector T cell
Treg	Regulatory T cell
Tem	Effector memory T cell
Tcm	Central memory T cell

## References

[1] Hosain M.Z., Kabir S.M.L., Kamal M.M. (2021). Antimicrobial Uses for Livestock Production in Developing Countries. Vet. World.

[2] Turner M.D., Nedjai B., Hurst T., Pennington D.J. (2014). Cytokines and Chemokines: At the Crossroads of Cell Signalling and Inflammatory Disease. Biochim. Biophys. Acta Mol. Cell Res..

[3] Takeuchi O., Akira S. (2010). Pattern Recognition Receptors and Inflammation. Cell.

[4] Rahman T., Das A., Abir M.H., Nafiz I.H., Mahmud A.R., Sarker M.R., Emran T.B., Hassan M.M. (2023). Cytokines and Their Role as Immunotherapeutics and Vaccine Adjuvants: The Emerging Concepts. Cytokine.

[5] Charerntantanakul W. (2020). Adjuvants for Swine Vaccines: Mechanisms of Actions and Adjuvant Effects. Vaccine.

[6] Blecha F., Reddy D.N., Chitko-McKown C.G., McVey D.S., Chengappa M.M., Goodband R.D., Nelssen J.L. (1995). Influence of Recombinant Bovine Interleukin-1β and Interleukin-2 in Pigs Vaccinated and Challenged with *Streptococcus suis*. Vet. Immunol. Immunopathol..

[7] Zhang L.-H., Tian X.-S., Guo Y., Zhou F.-Z., Meng M.-J. (2006). Effect of Transgenic Expression of Porcine Interleukin-6 Gene and CpG Sequences on Immune Responses of Newborn Piglets Inoculated with Pseudorabies Attenuated Vaccine. Res. Vet. Sci..

[8] Elias J.A., Zitnik R.J. (1992). Cytokine-Cytokine Interactions in the Context of Cytokine Networking. Am. J. Respir. Cell Mol. Biol..

[9] Ahlers J.D., Belyakov I.M., Matsui S., Berzofsky J.A. (2001). Mechanisms of Cytokine Synergy Essential for Vaccine Protection against Viral Challenge. Int. Immunol..

[10] Tang D., Liu J., Li C., Zhang H., Ma P., Luo X., Zeng Z., Hong N., Liu X., Wang B. (2014). Positive Effects of Porcine IL-2 and IL-4 on Virus-Specific Immune Responses Induced by the Porcine Reproductive and Respiratory Syndrome Virus (PRRSV) ORF5 DNA Vaccine in Swine. J. Vet. Sci..

[11] Dougan M., Dranoff G., Dougan S.K. (2019). GM-CSF, IL-3, and IL-5 Family of Cytokines: Regulators of Inflammation. Immunity.

[12] Hawley R.J., Abraham S., Akiyoshi D.E., Arduini R., Denaro M., Dickerson M., Meshalum D.H., Monroy R.L., Schacter B.Z., Rosa M.D. (1997). Xenogeneic Bone Marrow Transplantation: I. Cloning, Expression, and Species Specificity of Porcine IL-3 and Granulocyte-Macrophage Colony-Stimulating Factor. Xenotransplantation.

[13] Andrew M., Morris K., Coupar B., Sproat K., Oke P., Bruce M., Broadway M., Morrissy C., Strom D. (2006). Porcine Interleukin-3 Enhances DNA Vaccination against Classical Swine Fever. Vaccine.

[14] Chen D., Tang T.-X., Deng H., Yang X.-P., Tang Z.-H. (2021). Interleukin-7 Biology and Its Effects on Immune Cells: Mediator of Generation, Differentiation, Survival, and Homeostasis. Front. Immunol..

[15] Ueha S., Kitazawa H., Tomioka Y., Kawai Y., Saito T., Itoh T. (2001). cDNA Cloning and Expression of Swine IL-7 from Neonatal Intestinal Epithelium1. Biochim. Biophys. Acta Gene Struct. Expr..

[16] Perera P.-Y., Lichy J.H., Waldmann T.A., Perera L.P. (2012). The Role of Interleukin-15 in Inflammation and Immune Responses to Infection: Implications for Its Therapeutic Use. Microbes Infect..

[17] Zambello R., Facco M., Trentin L., Sancetta R., Tassinari C., Perin A., Milani A., Pizzolo G., Rodeghiero F., Agostini C. (1997). Interleukin-15 Triggers the Proliferation and Cytotoxicity of Granular Lymphocytes in Patients With Lymphoproliferative Disease of Granular Lymphocytes. Blood.

[18] Dinarello C.A. (2007). Historical Insights into Cytokines. Eur. J. Immunol..

[19] Eyerich K., Dimartino V., Cavani A. (2017). IL-17 and IL-22 in Immunity: Driving Protection and Pathology. Eur. J. Immunol..

[20] Sonnenberg G.F., Fouser L.A., Artis D. (2011). Border Patrol: Regulation of Immunity, Inflammation and Tissue Homeostasis at Barrier Surfaces by IL-22. Nat. Immunol..

[21] Zheng Y., Valdez P.A., Danilenko D.M., Hu Y., Sa S.M., Gong Q., Abbas A.R., Modrusan Z., Ghilardi N., de Sauvage F.J. (2008). Interleukin-22 Mediates Early Host Defense against Attaching and Effacing Bacterial Pathogens. Nat. Med..

[22] Geginat J., Sallusto F., Lanzavecchia A. (2001). Cytokine-Driven Proliferation and Differentiation of Human Naive, Central Memory, and Effector Memory CD4+ T Cells. J. Exp. Med..

[23] Lin S., Yang X., Liang D., Zheng S.G. (2014). Treg Cells: A Potential Regulator for IL-22 Expression?. Int. J. Clin. Exp. Pathol..

[24] Jeron A., Hansen W., Ewert F., Buer J., Geffers R., Bruder D. (2012). ChIP-on-Chip Analysis Identifies IL-22 as Direct Target Gene of Ectopically Expressed FOXP3 Transcription Factor in Human T Cells. BMC Genom..

[25] Underdown B.J., Schiff J.M. (1986). Immunoglobulin A: Strategic Defense Initiative at the Mucosal Surface. Annu. Rev. Immunol..

[26] Macpherson A.J., McCoy K.D., Johansen F.-E., Brandtzaeg P. (2008). The Immune Geography of IgA Induction and Function. Mucosal Immunol..

[27] Martinoli C., Chiavelli A., Rescigno M. (2007). Entry Route of Salmonella Typhimurium Directs the Type of Induced Immune Response. Immunity.

